# Chicago Mortality Report, for Feb. 1874

**Published:** 1874-04

**Authors:** 


### *Chicago Mortality Report for February, 1874.*

MORTALITY IN MONTH OF FEBRUARY, 1874.

| | |
|---|---|
| Accident, under narcotic | 1 |
| " " tanzy | 1 |
| " by railroad | 6 |
| " by scalding | 2 |
| Abdomen, congestion of | 1 |
| Anæmia | 1 |
| Apoplexy | 6 |
| Asthma | 3 |
| Bowels, congestion of | 1 |
| Brain, congestion of | 4 |
| " inflammation of | 4 |
| " softening of | 1 |
| Bronchitis | 17 |
| " capillary | 3 |
| " chronic | 2 |
| Cancer | 1 |
| " of breast | 1 |
| " of bowels | 1 |
| " of liver | 1 |
| " of stomach | 1 |
| " of uterus | 2 |
| Child-birth | 1 |
| Cholera Morbus | 1 |
| Chest, inflammation of | 1 |
| Consumption | 54 |
| Convulsions | 66 |
| Croup | 3 |
| " membranous | 1 |
| Cyanosis | 1 |
| Debility, general | 6 |
| Diphtheria | 4 |
| Dropsy, general | 2 |
| " of chest | 2 |
| Dysentery, chronic | 1 |
| Embolism | 2 |
| Exophthalmia | 1 |
| Enteritis | 6 |
| Exposure | 1 |
| Entero Colitis | 1 |
| Epilepsy | 1 |
| Erysipelas | 9 |
| Fever, intermittent | 1 |
| " puerperal | 10 |
| " scarlet | 1 |
| " " malignant | 1 |
| " typhoid | 11 |
| Gastritis | 2 |
| Heart, disease of | 4 |
| " paralysis | 1 |
| " aneurism of | 1 |
| " organic disease of | 2 |
| " hypertrophy of | 2 |
| " valvular disease of | 5 |
| Hemorrhage, cerebral | 1 |
| Hernia, strangulated | 1 |
| Hepatitis | 2 |
| Hydrocephalus | 7 |
| Inanition | 9 |
| Intemperance | 3 |
| Kidneys, Bright's disease of | 4 |
| " inflammation of | 2 |
| Laryngitis | 1 |
| Liver, cirrhosis of | 1 |
| " and kidneys, fatty degeneration of | 1 |
| Lungs, abscess of | 1 |
| " congestion of | 3 |
| " emphysema of | 1 |

| | |
|---|---|
| Lungs, hemorrhage of | 2 |
| Malformation | 1 |
| Metritis | 1 |
| " puerperal | 3 |
| Meningitis | 9 |
| " cerebro spinal | 9 |
| " tubercular | 1 |
| Mouth, canker sore | 1 |
| Nervous prostration | 1 |
| Nutrition impaired | 1 |
| Old Age | 17 |
| Orchitis | 1 |
| Œsophagitis | 1 |
| Paralysis | 3 |
| Parotitis | 3 |
| Pelvic Cellulitis | 1 |
| Pericarditis | 3 |
| Peritonitis | 6 |
| " puerperal | 3 |
| Pneumonia | 48 |
| " typhoid | 4 |
| Pleurisy | 7 |
| Pyæmia | 1 |
| Rheumatism | 1 |
| " inflammatory | 2 |
| Scrofula | 1 |
| Septicæmia | 1 |
| Small-Pox | 11 |
| Spinal Cord, disease of | 1 |
| Stomach, ulceration of | 1 |
| Suicide, drowning | 1 |
| " cutting throat | 1 |
| " hanging | 1 |
| Tabes Mesenterica | 7 |
| Teething | 4 |
| " and complication | 2 |
| Uterus, hemorrhage of | 3 |
| Vertebra, fracture of | 1 |
| Vitality deficient | 1 |
| Whooping Cough | 4 |
| Total | 472 |

Premature births, 15 ; Still births, 57. Total 72

COMPARISON.

| | | |
|---|---|---|
| Deaths in month of February, 1874 | | 472 |
| " " January, 1874 | | 553 |
| Decrease | 81 | |
| Deaths in month of February, 1873 | | 586 |
| Decrease | 114 | |

AGES.

| | |
|---|---|
| Under one year | 169 |
| One year to two | 33 |
| Two years to three | 10 |
| Three " " four | 5 |
| Four " " five | 4 |
| Five " " ten | 13 |
| Ten " " twenty | 29 |
| Twenty " " thirty | 59 |
| Thirty " " forty | 51 |
| Forty years to fifty | 39 |
| Fifty " " sixty | 18 |
| Sixty " " seventy | 18 |
| Seventy " " eighty | 19 |
| Eighty " " ninety | 3 |
| Ninety " " one hundred | -- |
| One hundred and upwards | 2 |
| Total | 472 |

| | |
|---|---|
| Colored | 7 |
| White | 465 |
| Total | 472 |
| Males | 236 |
| Females | 236 |
| Total | 472 |

Married, 156 ; Single, 316. Total 472

NATIVITIES.

| | |
|---|---|
| Austria | 1 |
| Bohemia | 7 |
| Canada | 5 |
| Native—Chicago | 44 |
| Foreign, " | 158 |
| United States, other parts | 98 |
| England | 6 |
| France | 1 |
| Denmark | 4 |
| Germany | 66 |
| Holland | 2 |
| Ireland | 53 |
| Norway | 5 |
| Scotland | 8 |
| Sweden | 9 |
| Switzerland | 1 |
| Unknown | 4 |
| Total | 472 |

Deaths daily, 17. Mean thermometer, 32.4°. Rain fall, 1.61 inches.

MORTALITY BY WARDS, ETC., FEBRUARY, 1874.

| Wards. | No. Deaths. | Pop. in 1872. | Percentage. |
|---|---|---|---|
| 1 | 4 | 398 | one death in ---- |
| 2 | 3 | 2,174 | " " " ---- |
| 3 | 18 | 19,157 | " " " 1,064 |
| 4 | 10 | 16,832 | " " " 1,683 |
| 5 | 11 | 18,564 | " " " 1,688 |
| 6 | 57 | 31,371 | " " " 550 |
| 7 | 40 | 27,644 | " " " 691 |
| 8 | 30 | 30,253 | " " " 1,008 |
| 9 | 26 | 30,032 | " " " 1,155 |
| 10 | 14 | 16,624 | " " " 1,189 |
| 11 | 18 | 18,340 | " " " 1,019 |
| 12 | 11 | 20 876 | " " " 1,898 |
| 13 | 20 | 14,636 | " " " 732 |
| 14 | 11 | 15,892 | " " " 1,445 |
| 15 | 52 | 40,047 | " " " 770 |
| 16 | 32 | 19,099 | " " " 597 |
| 17 | 21 | 17,513 | " " " 834 |
| 18 | 23 | 19,977 | " " " 868 |
| 19 | 6 | 2,944 | " " " ---- |
| 20 | 7 | 5,023 | " " " ---- |
| | 414 | Ratio of deaths to population in 1872, one death in 778½. | |

| | | | |
|---|---|---|---|
| No. deaths in Wards | 414 | Mercy Hospital | 4 |
| Accidents | 10 | St. Luke's Hospital | 1 |
| Bethel Home | 1 | St. Joseph's " | 3 |
| County Hospital | 17 | Suicides | 3 |
| Foundlings' Home | 16 | | |
| Hospital for Wives and Children | 1 | Total | 472 |
| Protestant Orphan Asylum | 2 | | |

CASES OF SMALL-POX REPORTED DURING FEBRUARY, 1874.

| Ward. | No. Cases. | Ward. | No. Cases. | Ward. | No. Cases. |
|---|---|---|---|---|---|
| 1 | -- | 8 | -- | 15 | 9 |
| 2 | -- | 9 | 1 | 16 | 6 |
| 3 | -- | 10 | -- | 17 | 1 |
| 4 | -- | 11 | 1 | 18 | 5 |
| 5 | -- | 12 | -- | 19 | 1 |
| 6 | -- | 13 | -- | 20 | -- |
| 7 | 8 | 14 | 1 | Recent arrival, | 1 |
| Total | | | | | 34 |

| | |
|---|---|
| Cases reported during February, 1874 | 34 |
| " " " January, 1874 | 79 |
| Decrease | 45 |

| | |
|---|---|
| Cases reported during February, 1873 | 161 |
| Decrease | 127 |

